# Haplotype network branch diversity, a new metric combining genetic and topological diversity to compare the complexity of haplotype networks

**DOI:** 10.1371/journal.pone.0251878

**Published:** 2021-06-30

**Authors:** Eric Garcia, Daniel Wright, Remy Gatins, May B. Roberts, Hudson T. Pinheiro, Eva Salas, Jei-Ying Chen, Jacob R. Winnikoff, Giacomo Bernardi

**Affiliations:** 1 Department of Ecology and Evolutionary Biology, University of California Santa Cruz, Santa Cruz, California, United States of America; 2 Department of Biological Sciences, Old Dominion University, Norfolk, Virginia, United States of America; 3 California Academy of Science, San Francisco, California, United States of America; 4 Department of Biology, Cabrillo College, Aptos, California, United States of America; 5 Monterey Bay Aquarium Research Institute, Moss Landing, California, United States of America; Florida Institute of Technology, UNITED STATES

## Abstract

A common way of illustrating phylogeographic results is through the use of haplotype networks. While these networks help to visualize relationships between individuals, populations, and species, evolutionary studies often only quantitatively analyze genetic diversity among haplotypes and ignore other network properties. Here, we present a new metric, haplotype network branch diversity (*HBd*), as an easy way to quantifiably compare haplotype network complexity. Our metric builds off the logic of combining genetic and topological diversity to estimate complexity previously used by the published metric haplotype network diversity (*HNd*). However, unlike *HNd* which uses a combination of network features to produce complexity values that cannot be defined in probabilistic terms, thereby obscuring the values’ implication for a sampled population, *HBd* uses frequencies of haplotype classes to incorporate topological information of networks, keeping the focus on the population and providing easy-to-interpret probabilistic values for randomly sampled individuals. The goal of this study is to introduce this more intuitive metric and provide an R script that allows researchers to calculate diversity and complexity indices from haplotype networks. A group of datasets, generated manually (model dataset) and based on published data (empirical dataset), were used to illustrate the behavior of *HBd* and both of its terms, haplotype diversity, and a new index called branch diversity. Results followed a predicted trend in both model and empirical datasets, from low metric values in simple networks to high values in complex networks. In short, the new combined metric joins genetic and topological diversity of haplotype networks, into a single complexity value. Based on our analysis, we recommend the use of *HBd*, as it makes direct comparisons of network complexity straightforward and provides probabilistic values that can readily discriminate situations that are difficult to resolve with available metrics.

## Introduction

In the past decades, the use of molecular data has allowed evolutionary, ecological, and conservation questions to be applied to non-model organisms in natural settings [1]. One way of illustrating molecular data for phylogeographic or intraspecific studies is the use of haplotype networks. These networks help to visualize relationships between individuals, populations, and species intuitively, revealing insights about migration, population structure, and speciation [2–4]. While estimating genetic diversity from haplotype networks is common, authors often rely on unquantified topological patterns (such as distributions of haplotypes across topologies) to make additional inferences since the combined genetic and topological components of haplotype network make quantitative comparisons difficult [2–4]. Thus, the implementation of standardized, quantitative methods of comparing haplotype networks that include topological features, will be constructive at a time when comparative phylogeography is becoming an increasingly useful tool to analyze complex geographic patterns of populations from multiple species in an ever-changing environment [4–7].

A variety of network features explained by graph theory such as node degrees, clustering coefficient, centralities, link prediction, and network density, among others, can be exploited to study the evolutionary inter-relationships between individuals illustrated in haplotype networks [8]. However, due to the paucity of interdisciplinary practices, such information is often overlooked in evolutionary studies even when this can be useful to quantifiably compare populations [8]. The introduction of the haplotype network diversity (*HNd*) metric, henceforth referred to as haplotype network node diversity, was a recent attempt to utilize network properties to quickly compare haplotype networks [3]. In that study, values of *HNd* (which incorporate haplotype and topological diversity of networks) were used to explore the correlation of endemism and genetic signatures of Galápagos fishes and test predictions of population structure in endemic, insular, and widely-distributed species [3]. The premise for the introduction of the *HNd* metric was a need for a single value to describe the complexity of haplotype networks in terms of their genetic and topological diversity, that would be simple and intuitive [3]. Since most scientists that use haplotype networks are usually also familiar with the concept and values of haplotype diversity (*Hd*), the intent was to produce a value similar in concept to *Hd* that would, in addition, capture the topological diversity of the haplotype network.

The *Hd* metric was first introduced by Nei in 1987, as the probability that two randomly sampled haplotypes are different [9]. While a number of approaches have refined the theoretical framework and the actual implementation on haplotype networks [10, 11], *Hd*, which varies between zero (all haplotypes are identical) and one (all haplotypes are different), has remained the metric of choice, and has been universally used to quickly and simply describe how genetically diverse populations are. In fact, the original description has been cited nearly 10,000 times since its publication according to the Web of Science (WoS) and remains current to this day [12]. Yet, *Hd* does not take network topology into consideration and its use for comparing networks is limited because very different haplotype networks can have the same *Hd*. The goal of the original description of *HNd* was therefore to provide a metric akin to *Hd* that would, in a single value, incorporate genetic and topological diversities and be familiar and intuitive to the users.

Nevertheless, the original description did not elaborate on the *HNd* approach itself, describe the method in detail, nor discuss the major differences between the methodology calculating *Hd* and the component of *HNd* intended to emulate *Hd*. In this study, we explain in detail the method used to obtain *HNd*, its principles, and pitfalls. We then introduce branch diversity (*Bd*), a new index that mirrors the logic used by *Hd* to estimate topological diversity of networks. Finally, we combined *Bd* with *Hd* into a single complexity metric, haplotype network branch diversity (*HBd*), which provides probabilistic values useful for comparing networks in phylogeographic studies. In addition, we include the R script called *HapNetComplexity* in the Supporting Information for ease of computing the metrics. Branch and haplotype network branch diversities can be calculated for any network regardless of the method and graphical tool used for construction [10, 13–21]. However, we illustrate the behavior of the new metrics using distance-based networks built with the package pegas [19] in R [22], as it remains a commonly used approach in population genetic studies [13, 23].

## Materials and methods

**Fig 1:**
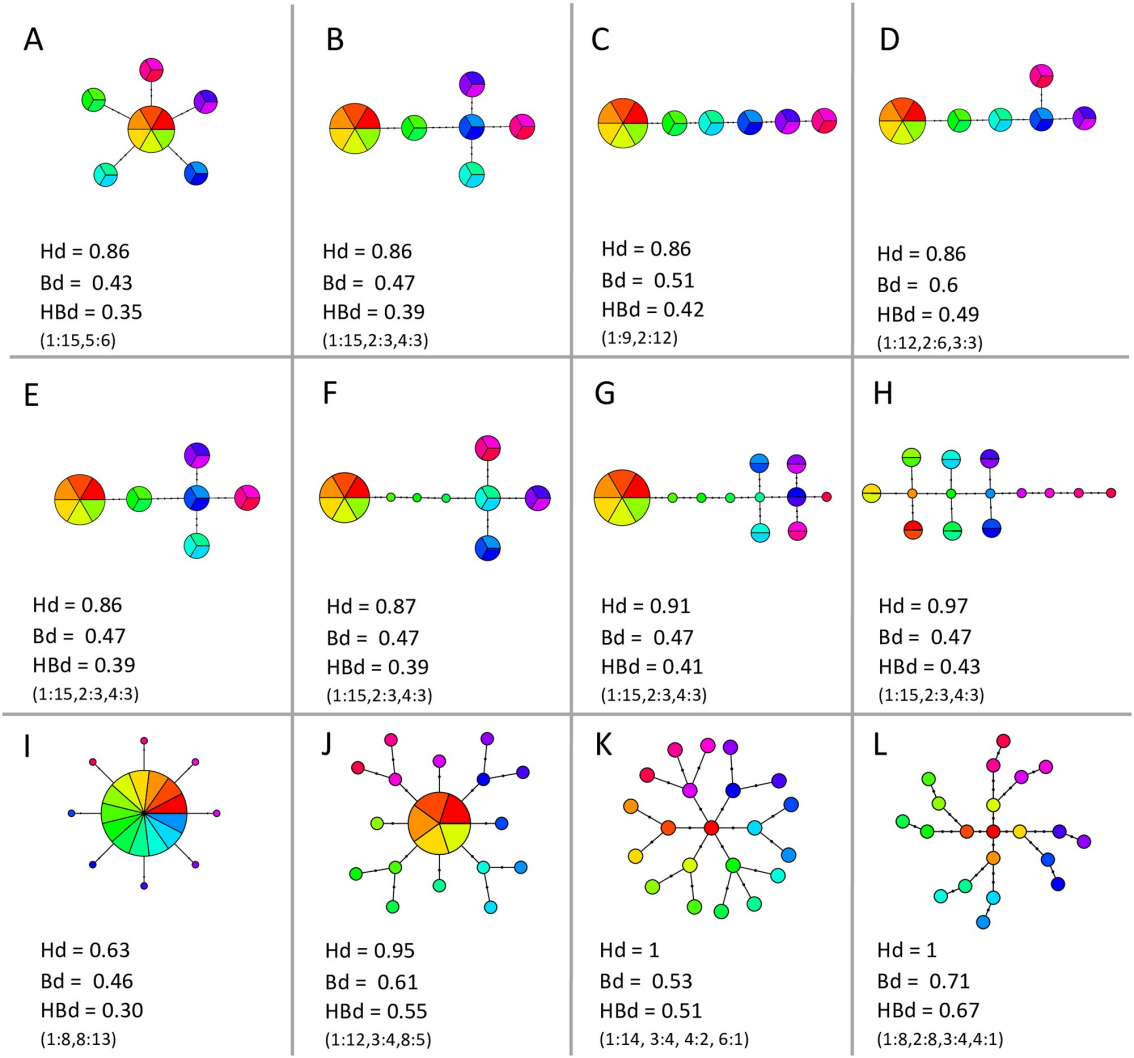
Haplotype networks and diversity indices for manually generated sequence datasets. Values for haplotype diversity (*Hd*), branch diversity (*Bd*), and haplotype network branch diversity (*HBd*), are given for each haplotype network. Colors represent individuals since each individual was intentionally set to represent a distinct population. For each network, haplotype classes (*Hc*) are represented in parenthesis with pairs of numbers where the class’ number of branches (*nbHc)* and individuals (*niHc*) are presented to the left and right of a colon, respectively. For example, the haplotype network in Panel A is made up of two haplotype classes, thus (1:15, 5:6) represents that there are 15 individuals within the 1-branch haplotype class and 6 individuals within the 5-branch haplotype class. This breakdown indicates the number of haplotype classes and the frequency-evenness among them, components which directly affect *Bd* and subsequently, *HBd*. All panels show datasets comprising 21 individuals and ranging from 6 to 21 haplotypes. Top panels (A, B, C, and D) illustrate four simple network configurations with six haplotypes that maintain *Hd* constant but that can be differentiated by *Bd* and *HBd*. Middle panels (E, F, G, and H) show variation in network configuration that maintains *Bd* constant but increases *Hd* from left to right. Bottom panels show more complex haplotype networks with 9, 17, 21, and 21 haplotypes, in panels I, J, K, and L, respectively, where *Bd* provides a larger margin to make comparisons than *Hd*, particularly between panels with equal *Hd* values (H and L). Additional dataset information for each panel is given in Table 1. Sequence and site files for all panels can be found in (S1–S13 Files).

**Fig 2:**
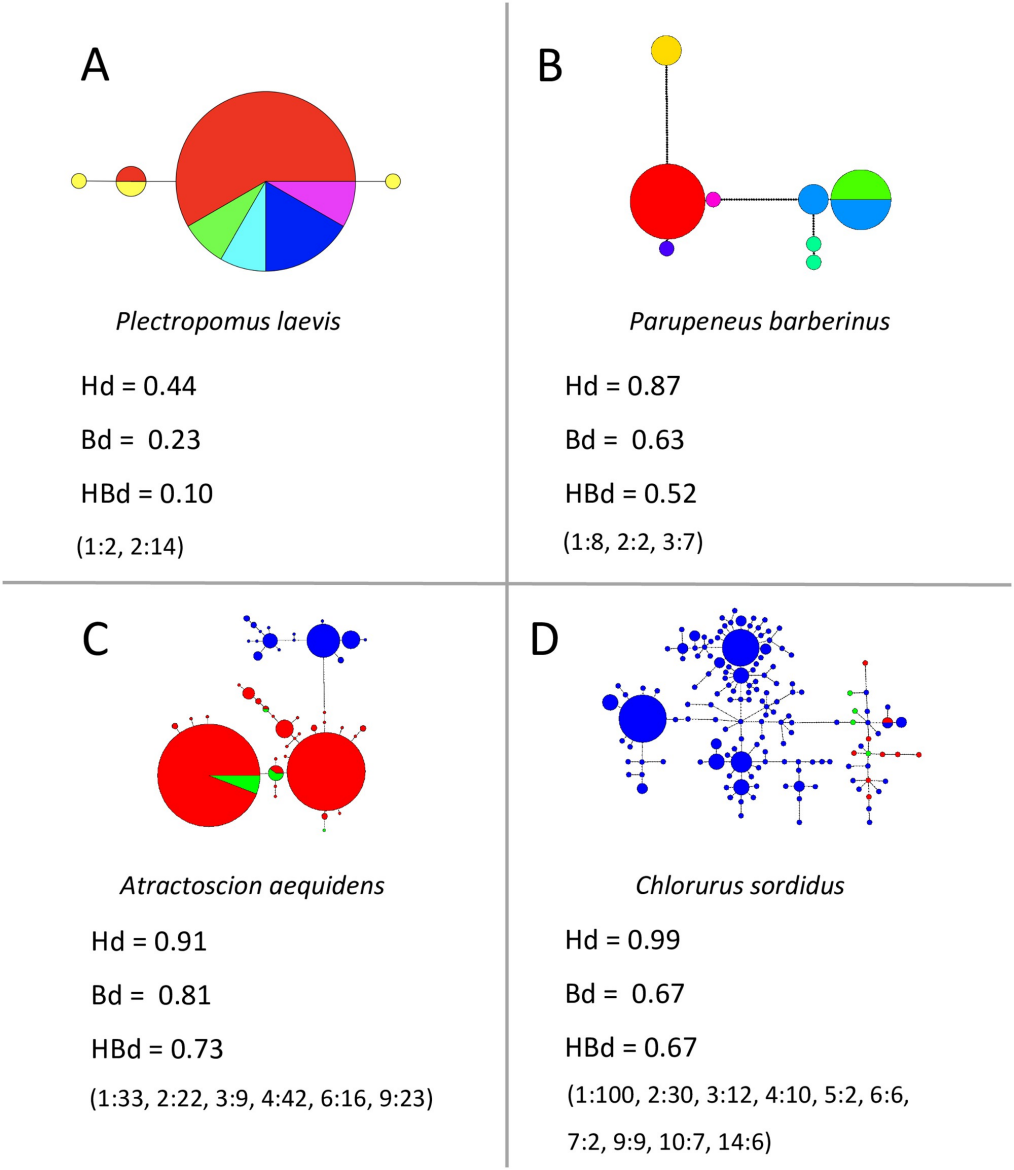
Haplotype networks and diversity indices for published sequence datasets. Colors represent sampled populations. All datasets (described in the Methods section) are based on CO1 or d-loop sequences of fish species shown in italics and were chosen to represent real scenarios with different levels of network complexity (A and B [26]; C [25]; and D [24]). Values for haplotype diversity (*Hd*), branch diversity (*Bd*) and haplotype network branch diversity (*HBd*) are given for each haplotype network. Similar to Fig 1, haplotype classes (*Hc*) are represented in parenthesis with pairs of numbers where the class’ number of branches (*nbHc*) and individuals (*niHc*) are presented to the left and right of a colon, respectively. Additional values referring to these networks are given in Table 1. Sequence and site files for all panels can be found in (S14–S21 Files).

Separate datasets that cover a range of network topologies and number of haplotypes were used to illustrate the behavior of new metrics in comparison to *Hd* (See Box 1 for metric and variable definitions). The first dataset was generated manually to show results from simple to more complex hypothetical networks (Fig 1). The second dataset, taken from GenBank and compiled in fasta format, used empirical networks also representing a range of complexities from previously published data [24–26] (Fig 2). Our first objective with these two datasets was to depict the variability of network complexity by displaying how different configurations can affect genetic and topological diversity estimates alike. We also used these datasets to showcase the ability of our new metric, *Bd*, to quantitatively discriminate simple and complex networks even when *Hd* remains constant. A third and final dataset was created manually to demonstrate the various properties of *Bd* (see Box 2 and Supporting information). All data files are available as (S1–S29 Files for the first, second and final dataset, respectively). We begin exploring the presented networks by computing *Hd* and *Bd*. Then, we combine these indices to calculate our other new metric, *HBd*, in order to compare the complexity of each haplotype network. All metrics were computed with the provided R script *HapNetComplexity* (S30 File), and range from zero to one making comparisons and replication of results straightforward. We would like to note that π (nucleotide diversity), another standard metric that captures the genetic distance between sequences, was intentionally not included in our calculations because it does not relate to network topology, and in some cases negates, or overwhelms other factors. However, π is also included in the R script for comparison purposes.

**Box 1. Metric and variable definitions**

**nH** Number of haplotypes in a network
**Hc** Haplotype class (*Hc)* is a classification of haplotypes according to their number of subtending branches in a network. Haplotypes with the same number of branches are classified into a unique group or haplotype class. Properties of haplotype classes include the number of branches (*nbHc)*, number of haplotypes (*nhHc)* and number of individuals (*niHc)*, all of which, might or might not be different within each class
**nHc** Number of haplotypes classes in a haplotype network
**Hd** Haplotype diversity (*Hd*) is the probability that two randomly selected individuals from a population will have different haplotypes
**Nd** Node diversity (*Nd)* is an adjusted node degree estimate of the haplotype classes in a network
**HNd** Haplotype network diversity (*HNd*) is a metric that estimates complexity of haplotype networks by combing *Hd* and *Nd*
**Bd** Branch diversity (*Bd)* is the probability that two randomly selected individuals from a population will have haplotypes with different numbers of branches in a network (i.e. unique haplotype classes)
**HBd** Haplotype network branch diversity (*HBd)* is a metric that estimates complexity of haplotype networks by combing *Hd* and *Bd*, both of which are frequencies. Therefore, *HBd* is the probability that two randomly selected individuals from a population will have distinct haplotypes with different numbers of branches in a haplotype network

*Note: Nd and HNd cannot be defined in probabilistic terms

**Box 2. Properties of Branch Diversity (*Bd*)**

1. ***Bd*=0 when there is only one class of haplotype regardless of its frequency (*niHc* or the number of individuals in class).** Haplotype classes are formed by haplotypes with the same number of subtending branches. Thus, *Bd=0* when the entire sequence pool contains only one haplotype, for which a network cannot be built, two haplotypes, in which case both haplotypes have only one branch, or more than two haplotypes forming a circular network where all haplotypes have exactly two branches. In all of these cases, diversity is zero since there is only one haplotype class.
2. ***Bd* reaches the theoretical value of 1 when every individual forms a different haplotype class (i.e. haplotypes with a unique number of branches).** However, this cannot be accomplished as more haplotypes than haplotype classes are needed to create a network. *Bd* approaches 1 in networks exhibiting large numbers of haplotype classes with similar or even frequencies.
3. ***Bd* increases with increasing number of haplotype classes.**
4. ***Bd* increases with higher frequency-evenness among haplotype classes (more even number of individuals among classes).**
5. ***Bd* decreases with increasing number of individuals unless these add new haplotype classes.** This occurs because these sequences do not add branch diversity but duplicate that already existing in a network.

*See Supporting Figs and Tables for demonstration of properties

### Haplotype diversity

Each of the discussed complexity metrics (*HNd* and *HBd*) considers sequence variation in networks by including *Hd* [9]. As a reminder, *Hd* is the probability of randomly drawing two different haplotypes from the population and values range from zero, where all haplotypes are the same, to one, where each individual has a different haplotype [9].

The formula for haplotype diversity is:

$$Hd\;=\;\left(1-\sum f_{h}^{2}\right)*\left(\frac{n}{n-1}\right)$$ (1)

where *f*_h_ is the frequency of haplotype *h* in the population and *n* is the total number of individuals.

### Node diversity

The haplotype network node diversity (*HNd*) metric was first described in a study that compared haplotype networks for Galápagos fishes [3]. The method combined two indices, *Hd* and node diversity (*Nd*), to describe haplotype networks. Node diversity incorporates the topology of a network using an approach intended to emulate the calculation of *Hd*.

For each network, a list of haplotypes is established and the number of subtending branches in each haplotype is counted. Haplotypes with the same number of branches are classified into a unique group called haplotype class (*Hc)*. Then, an adjusted degree distribution is calculated as shown below (modified from Bernardi *et al*. 2014):

$$Nd\;=\;\left(1-\;\sum \left[nhHc*{\left(\frac{nbHc}{nH}\right)}^{2}\right]\right)*\left(\frac{nH}{nH-1}\right)$$ (2)

where *nhHc* is the number of haplotypes in haplotype class *Hc*, *nbHc* is the number of branches in haplotype class *Hc*, and *nH* is the total number of haplotypes in the network (note that *nH* is also denoted as *nu* in Bernardi *et al*. 2014).

As an example, we use the dataset illustrated in Fig 1, Panel B. In a network with a total of six haplotypes, one haplotype is subtended by four branches, another by two branches, and four haplotypes have one branch subtending them. Therefore, there are three classes of haplotypes, with one, two, and four branches. Furthermore, these three classes hold four, one, and one haplotypes, respectively. In this example, the above formula results in:

$$Nd\;=\;\left(1-\left(\left[4*{\left(\frac{1}{6}\right)}^{2}\right]+\left[1*{\left(\frac{2}{6}\right)}^{2}\right]+\left[1*{\left(\frac{4}{6}\right)}^{2}\right]\right)\right)*\left(\frac{6}{5}\right)$$

Nd=0.4

While this metric is indicative of haplotype network complexity, it is not analogous to the way *Hd* is calculated because the adjusted degree distribution is calculated by dividing the number of branches in a given haplotype class (1-branch, 2-branch, or 4-branch, in this example) by the total number of haplotypes in the network (6 haplotypes) and as such, does not strictly conform to the definition of a frequency (see below for more detail). Moreover, combining *Nd* and *Hd* values to calculate *NHd* does not result in probabilistic values since a frequency is not calculated in *Nd*. We therefore build upon the idea from which *Nd* was initially formulated to explore a new way (*Bd)* to estimate topological diversity that would be comparable to *Hd* and that can properly be merged into a single probabilistic value.

### Branch diversity

In order to be consistent in our calculation of frequencies, we introduce a new metric, branch diversity (*Bd*). Similar to *Nd*, the first step is to categorize all haplotypes by the number of branches that stem from them into haplotype classes (*Hc*). Unlike *Nd*, however, frequencies of haplotype classes are calculated by dividing the number of individuals in each class by the total number of individuals in your sample, rather than dividing the number branches by the number of haplotypes, thus resulting in actual frequencies that are consistent with the calculation of *Hd*. Following the same logic as *Hd*, *Bd* can be defined as the probability that two randomly selected individuals in a population will have haplotypes with different numbers of branches in a haplotype network (Box 1). Branch diversity ranges from zero, when there is only one haplotype class in a network, to one, when every individual produces a different haplotype class in a network.

Using the same example as above (Fig 1, Panel B), the network contains 21 individuals and 6 haplotypes that can be grouped into three different haplotype classes (1-branch, 2-branch and 4-branch). The haplotype classes with 1, 2, and 4 branches, contain 15, 3, and 3 individuals, respectively. We then calculate a frequency, similarly to how *Hd* is computed:

$$Bd\;=\;(1-\sum f_{Hc}^{2})*\left(\frac{n}{n-1}\right)$$ (3)

where *f*_*Hc*_ is the frequency of haplotype class *Hc* (i.e. *niHc*/*n* or the number of individuals in haplotype class *Hc* divided by the total number of individuals in the sample), and *n* is the total number of individuals analyzed. Using the same dataset as the previous example, the above formula results in:

$$Bd\;=\;\left(1-\left[{\left(\frac{15}{21}\right)}^{2}+{\left(\frac{3}{21}\right)}^{2}+{\left(\frac{3}{21}\right)}^{2}\right]\right)*\left(\frac{21}{20}\right)$$

Bd=0.47

### Haplotype network branch diversity

To illustrate the complexity of the haplotype networks, we combine the two indices of diversity, *Hd* and *Bd*, to obtain *HBd* as follows:

HBd=Hd*Bd

$$HBd\;=\;\left(1-\sum {\;f}_{h}^{2}\right)*\left(1-\sum {\;f}_{Hc}^{2}\right)*(\frac{n}{n-1})$$ (4)

where *HBd* is haplotype network branch diversity and the two main terms are haplotype diversity (*Hd*) and branch diversity (*Bd*).

### Computer script

The computer script *HapNetComplexity* was written in R [22] using the pegas package [19] as the main resource to produce haplotype networks. The script, available in the (S30 File) and from https://github.com/ericgarciaresearch/Haplotype-network-branch-diversity_HBd, allows for the easy construction of haplotype networks and the computation of all analyzed metrics.

## Results and discussion

In this study, we present the new metric haplotype network branch diversity (*HBd*), as a tool to quantitatively compare and illustrate the variability of haplotype network complexity. Haplotype network branch diversity is computed by combining the commonly quantified haplotype diversity (*Hd*), with the new index of the topological diversity of haplotype networks, branch diversity (*Bd*). While *Hd* calculates the genetic diversity of a population, *Bd* measures the diversity of the evolutionary interrelationships between the haplotypes observed in a population. All metrics, *Hd*, *Bd*, and *HBd*, vary between zero and one allowing for direct comparisons of diversity and complexity between networks.

The first manually generated model dataset consists of 21 individuals that partition into 6, 8, 9, 11, 14, 17, or 21 haplotypes, and 2, 3, or 4 classes of haplotypes (Fig 1 and Table 1). Haplotype diversity varies between 0.63 and 1.00, *Bd* varies between 0.43 and 0.71, and *HBd* varies between 0.3 and 0.67 (Fig 1 and Table 1). The top row of Fig 1 (Panels A-D) shows that even when *Hd* remains constant, *Bd* varies depending on the network topology, clearly illustrating its ability to distinguish between simple networks.

**Table 1:** Diversity indices for model (Fig 1) and published datasets (Fig 2).

**Panel**	**Model data**	***n***	***nH***	***nHc***	***Hd***	***Bd***	***HBd***
A	testing_A	21	6	2	0.86	0.43	0.35
B	testing_B	21	6	3	0.86	0.47	0.39
C	testing_C	21	6	2	0.86	0.51	0.42
D	testing_D	21	6	3	0.86	0.6	0.49
E	testing_E	21	6	3	0.86	0.47	0.39
F	testing_F	21	8	3	0.87	0.47	0.39
G	testing_G	21	11	3	0.91	0.47	0.41
H	testing_H	21	14	3	0.97	0.47	0.43
I	testing_I	21	9	2	0.63	0.5	0.3
J	testing_J	21	17	3	0.95	0.61	0.55
K	testing_K	21	21	4	1	0.53	0.51
L	testing_L	21	21	4	1	0.71	0.67
**Panel**	**Published data**	***n***	***nH***	***nHc***	***Hd***	***Bd***	***HBd***
A	*P*. *laevis*	16	4	2	0.44	0.23	0.1
B	*P*. *barberinus*	17	8	3	0.87	0.63	0.52
C	*A*. *aequidens*	145	48	6	0.91	0.81	0.73
D	*C*. *sordidus*	185	152	10	0.99	0.67	0.66

Columns from left to right: Panel and names of datasets corresponding to Figs 1 and 2, number of individuals (*n*), number of haplotypes (*nH*), number of haplotype classes (*nHc*), haplotype diversity (*Hd*), branch diversity (*Bd*), and haplotype network branch diversity (*HBd*).

Panels E-H of Fig 1, illustrate how networks with a similar topology, but increasing number of haplotypes, increase *Hd* and can maintain the same *Bd* values if the number of haplotype classes and their number of individuals are unchanged. Finally, in Panels I-L of Fig 1, *Bd* increases as topology becomes more compounded, and the number of haplotype classes increase from 2 to 4. In fact, the two main factors influencing *Bd* are the number of haplotype classes in a network and how evenly distributed the individuals are among these classes. Branch diversity increases when the number of haplotype classes also increases and with a more even number of individuals among the classes (see Box 2 and Supporting information for all *Bd* properties). This behavior results in *Bd* values that are low for simple networks and rise with increasing complexity. Unlike *Hd*, the multi-property character of *Bd* allows it to discriminate between networks with equal number of haplotypes or haplotype frequencies, whether these have simple (Fig 1, Panels A-D) or complex (Fig 1, Panels K-L) topologies. In contrast, *Hd* is able to capture variation in the number of haplotypes in situations when this does not affect *Bd*. Thus, combining *Hd* and *Bd* into *HBd* allows this metric to distinguish networks with the same number of haplotypes but diverse topologies (which keeps *Hd* constant but affects *Bd*; Fig 1, Panels A-D, K, L) and networks with similar topologies and diverging number of haplotypes (which influences *Hd* but it might not affect *Bd;* Fig 1, Panels G and H). Given that both *Hd* and *Bd* are calculated with frequencies, this comprehensive measurement of network complexity is provided as a single probabilistic value, the main goal of this study.

Similarly, the values of *Hd*, *Bd*, and *HBd* from networks of published sequence data (Fig 2) vary between 0.44–0.99, 0.23–0.81, and 0.1–0.73, respectively. Branch diversity and *HBd* span a wider range than *Hd*. The lowest values for all metrics are found within *Plectropomus laevis* (Fig 2, Panel A), a widespread grouper with a recent population expansion, which show only four haplotypes in its range [26]. The highest value for *Hd* was found in *Chlorurus sordidus* (Fig 2, Panel D), which exhibited a complex haplotype network driven by a large distribution associated with a recent history of repeated shifts between isolation and increased migration amongst populations [24]. In comparison, the highest *Bd* and *HBd* values are recorded in *Atractoscion aequidens* due to the combination of having many haplotype classes and these occurring at similar frequencies. This is a pelagic fish species, found along the coast of southwestern Africa, in which an ancient vicariant event has been proposed to explain two very distinct genetic lineages (shown in red and blue, respectively) [25]. It is worth noting that the network created by the *C*. *sordidus* dataset actually contains more haplotype classes than that of *A*. *aequidens* (10 vs 6), however, it also has a heavily skewed distribution of individuals as two of these classes alone (1-branch and 2-branch classes) hold more than 70% of all the individuals, which keeps *Bd* relatively low (Fig 2 and Table 1). Furthermore, while *Bd* is not directly affected by the actual number of branches in the different haplotype classes (just as *Hd* is not affected by the distance among haplotypes), the fact that more than half of the *C*. *sordidus* haplotypes were subtended by only one branch, limits the network from increasing its topological diversity, and ultimately dampens its overall complexity score (*HBd)*.

Ultimately, the complexity estimates of *HBd* simultaneously provide a measurement of the distinctness of individuals (as calculated by *Hd*) and the diversity of the interrelationships among them (as calculated by *Bd)*. For instance, *Hd* is relatively higher than *Bd* in populations where rare variants are prevalent. This scenario might arise as a result of, among others, a population expansion (as in *P*. *laevis*; Fig 2, Panel A), repeated cycles of isolation and secondary contacts (as in *C*. *sordidus*; Fig 2, Panel A), or a hypothetical bottleneck that produces a variety of haplotypes by chance (i.e. not heavily dominated by few haplotypes). In contrast, *Bd* is highest when a population conserves high connectivity among haplotypes (rare variants just represent another haplotype class) and classes have similar or equal frequencies. Yet, the more haplotypes present in a population the harder it becomes to maintain high connectivity and an even distribution of haplotypes across classes (as in *C*. *sordidus*). Whereas mechanisms that advocate the conservation of diversity such as random mating and balancing selection should help maintain high *Bd* values in a population, other processes such as assortative mating or directional selection are likely to decrease this measurement. In this way, *Hd* and *Bd* describe distinct properties of a population and can be used independently in evolutionary studies and conservation strategies with different goals. Compared to *Hd* or *Bd* alone, *HBd* provides a more holistic and conservative view of populations where high values indicate populations with high genetic diversity with well interconnected haplotypes that also include rare variants.

Inferring the evolutionary history of populations using haplotype networks presents several challenges including the difficulty comparing networks, network reticulations, alternative links, missing haplotypes, etc. Yet, solutions to these challenges are likely to be developed as interdisciplinary approaches become more frequent. This study provides a useful and simple tool to describe haplotype networks and streamline comparisons between network complexity, a property traditionally overlooked in evolutionary studies. The metric introduced herein simultaneously quantifies genetic and topological diversity of networks while also discriminating situations that are difficult to resolve with simpler available metrics. Furthermore, since every network is treated independently, our metric can be applied across haplotype building methods and alternate networks within a set of sequences. We therefore recommend the use of haplotype network branch diversity (*HBd*) as a single metric to describe and easily compare the complexity of different haplotype networks.

## Supporting information

S1 Fig Haplotype networks for manually generated datasets demonstrating branch diversity (*Bd*) properties 3 and 4 from Box 2. Values for haplotype diversity (*Hd*), branch diversity (*Bd*) and haplotype network branch diversity (*HBd*) are shown for each haplotype network. Colors represent individuals since each individual was set to represent a distinct population. For each network, haplotype classes (*Hc*) are represented in parenthesis with pairs of numbers where the number of branches (*nbHc*) and individuals within each class (*niHc*) are presented to the left and right of a colon, respectively. For instance, the network in Panel B contains two haplotype classes, a 1-branch class with 24 individuals and a 2-branch class with also 24 individuals. All networks contain the same total number of individuals (n = 48), range from 2 to 31 haplotypes, and are placed in order of increasing *Bd* from left to right. Top panels (A, B, C, and D) illustrate how increasing the number of haplotype classes, *nHc*, increases *Bd* values (property 3). The lower panels (E, F, G, and H) illustrate how increasing frequency-evenness among haplotype classes (i.e. maintaining the same number of haplotype classes and adjusting the number of individuals among classes, *niHc*) increases *Bd* (property 4). Additional dataset information for each panel is given in S1 Table. Sequence files for all panels can also be found in (S22–S29 Files).
(TIFF)

S2 Fig Regression plots demonstrating branch diversity (*Bd*) properties 3 and 5 from Box 2. Panel A shows *Bd* increase with increasing number of haplotype classes, *nHc*, in the form of an asymptotic curve as it approaches the value of 1 (property 3). The frequency of each class, *niHc*, here is held constant to isolate the effect of adding classes. Panel B illustrates how *Bd* decreases when the number of individuals (*n)* increases without adding new haplotype classes (i.e. increasing the frequency of existing haplotype classes, *niHc*) (property 5). The last occurs because these individuals do not add to branch diversity but replicate that already existing. The example in Panel B represents a simulation with a constant number of haplotype classes (5) and a range of 10 to 10x10^10^ individuals.
(TIFF)

S1 Table Demonstration of branch diversity (*Bd*) properties. (DOCX)

S2 Table Demonstration of branch diversity (*Bd*) properties. (DOCX)

S3 Table Demonstration of branch diversity (*Bd*) properties. (DOCX)

S1 File Testing_A.fasta. Sequence data for Fig 1, Panel A.
(FASTA)

S2 File Testing_B.fasta. Sequence data for Fig 1, Panel B.
(FASTA)

S3 File Testing_C.fasta. Sequence data for Fig 1, Panel C.
(FASTA)

S4 File Testing_D.fasta. Sequence data for Fig 1, Panel D.
(FASTA)

S5 File Testing_E.fasta. Sequence data for Fig 1, Panel E.
(FASTA)

S6 File Testing_F.fasta. Sequence data for Fig 1, Panel F.
(FASTA)

S7 File Testing_G.fasta. Sequence data for Fig 1, Panel G.
(FASTA)

S8 File Testing_H.fasta. Sequence data for Fig 1, Panel H.
(FASTA)

S9 File Testing_I.fasta. Sequence data for Fig 1, Panel I.
(CSV)

S10 File Testing_J.fasta. Sequence data for Fig 1, Panel J.
(FASTA)

S11 File Testing_K.fasta. Sequence data for Fig 1, Panel K.
(CSV)

S12 File Testing_L.fasta. Sequence data for Fig 1, Panel L.
(FASTA)

S13 File Sites_for_all_Fig1.csv. (CSV)

S14 File Plectropomus_laevis.fasta. Sequence data for Fig 2, Panel A.
(FASTA)

S15 File Plectropomus_laevis_sites. Sites for Fig 2, Panel A.
(CSV)

S16 File Parupeneus_barberinus.fasta. Sequence data for Fig 2, Panel B.
(FASTA)

S17 File Parupeneus_barberinus_sites. Sites for Fig 2, Panel B.
(CSV)

S18 File Atractoscion_aequidens.fasta. Sequence data for Fig 2, Panel C.
(FASTA)

S19 File Atractoscion_aequidens_sites. Sites for Fig 2, Panel C.
(FASTA)

S20 File Chlorurus_sordidus.fasta. Sequence data for Fig 2, Panel D.
(FASTA)

S21 File Chlorurus_sordidus_sites. Sites for Fig 2, Panel D.
(FASTA)

S22 File Testing_S1_A.fasta. Sequence data for S1 Fig, Panel A.
(FASTA)

S23 File Testing_S1_B.fasta. Sequence data for S1 Fig, Panel B.
(FASTA)

S24 File Testing_S1_C.fasta. Sequence data for S1 Fig, Panel C.
(FASTA)

S25 File Testing_S1_D.fasta. Sequence data for S1 Fig, Panel D.
(FASTA)

S26 File Testing_S1_E.fasta. Sequence data for S1 Fig, Panel E.
(R) Click here for additional data file.

S27 File Testing_S1_F.fasta. Sequence data for S1 Fig, Panel F.
(FASTA)

S28 File Testing_S1_G.fasta. Sequence data for S1 Fig, Panel G.
(FASTA)

S29 File Testing_S1_H.fasta. Sequence data for S1 Fig, Panel H.
(FASTA)

S30 File HapNetComplexity.R. R script to build haplotype networks and calculate analyzed metrics.
(R) Click here for additional data file.
