# Supplementary figures and images for: Haplotype network branch diversity, a new metric combining genetic and topological diversity to compare the complexity of haplotype networks
Source: PLoS One. 2021 Jun 30;16(6):e0251878. doi: 10.1371/journal.pone.0251878 (PMC8244886; doi:10.1371/journal.pone.0251878)

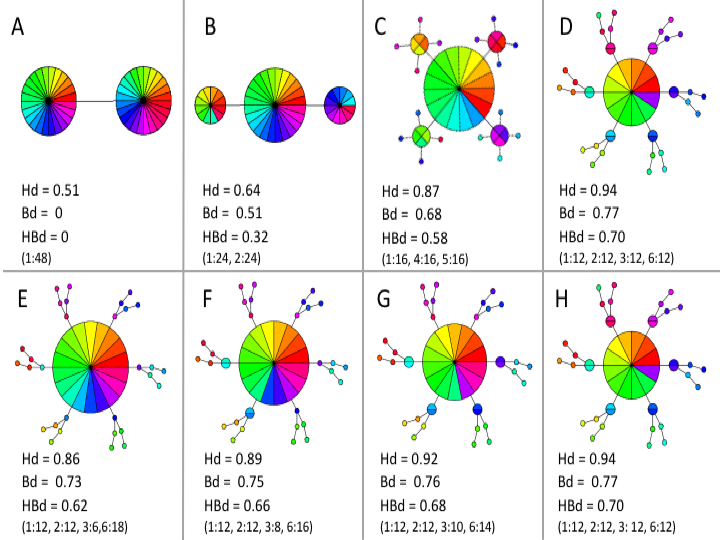

Supplement: S1 Fig — Values for haplotype diversity (Hd), branch diversity (Bd) and haplotype network branch diversity (HBd) are shown for each haplotype network. Colors represent individuals since each individual was set to represent a distinct population. For each network, haplotype classes (Hc) are represented in parenthesis with pairs of numbers where the number of branches (nbHc) and individuals within each class (niHc) are presented to the left and right of a colon, respectively. For instance, the network in Panel B contains two haplotype classes, a 1-branch class with 24 individuals and a 2-branch class with also 24 individuals. All networks contain the same total number of individuals (n = 48), range from 2 to 31 haplotypes, and are placed in order of increasing Bd from left to right. Top panels (A, B, C, and D) illustrate how increasing the number of haplotype classes, nHc, increases Bd values (property 3). The lower panels (E, F, G, and H) illustrate how increasing frequency-evenness among haplotype classes (i.e. maintaining the same number of haplotype classes and adjusting the number of individuals among classes, niHc) increases Bd (property 4). Additional dataset information for each panel is given in S1 Table. Sequence files for all panels can also be found in (S22–S29 Files). (TIFF) [file pone.0251878.s001.tiff]

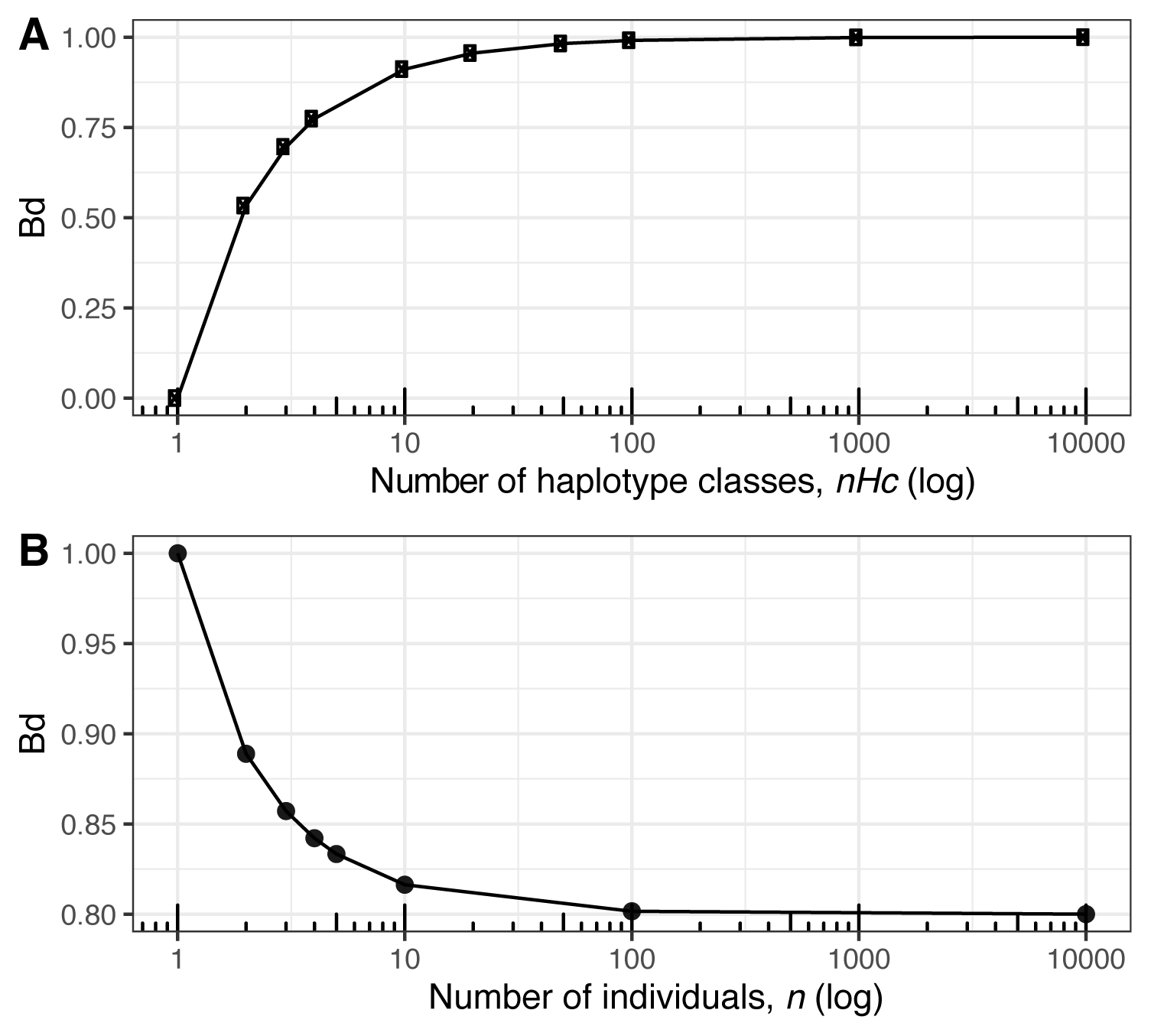

Supplement: S2 Fig — Panel A shows Bd increase with increasing number of haplotype classes, nHc, in the form of an asymptotic curve as it approaches the value of 1 (property 3). The frequency of each class, niHc, here is held constant to isolate the effect of adding classes. Panel B illustrates how Bd decreases when the number of individuals (n) increases without adding new haplotype classes (i.e. increasing the frequency of existing haplotype classes, niHc) (property 5). The last occurs because these individuals do not add to branch diversity but replicate that already existing. The example in Panel B represents a simulation with a constant number of haplotype classes (5) and a range of 10 to 10x1010 individuals. (TIFF) [file pone.0251878.s002.tiff]
