# Supplementary material for: Haplotype network branch diversity, a new metric combining genetic and topological diversity to compare the complexity of haplotype networks
Source: PLoS One. 2021 Jun 30;16(6):e0251878. doi: 10.1371/journal.pone.0251878 (PMC8244886; doi:10.1371/journal.pone.0251878)
Supplement: S1 Table — (DOCX) [file pone.0251878.s003.docx]

**S1 Table.** **Diversity indices for model data in S1 Fig.** Panels illustrate the effect of increasing the number of haplotype classes (property 3; Panels A-D) and the frequency-evenness among haplotype classes (even number of sequences) (property 4; Panels A-D) on *Bd*. First three columns represent the file and situation illustrated by each panel in S1 Fig; after, number of individuals (*n*), number of haplotypes (*nH*), number of haplotype classes (*nHc*), haplotype diversity (*Hd*), branch diversity (*Bd*), and haplotype network branch diversity (*HBd*).

| **Panel** | **File** | **Situation** | ***n*** | ***nH*** | ***nHc*** | ***Hd*** | ***Bd*** | ***HBd*** |
| --- | --- | --- | --- | --- | --- | --- | --- | --- |
| A | testing_S1_A | 1 haplotype class | 48 | 2 | 1 | 0.51 | 0 | 0 |
| B | testing_S1_B | 2 haplotype classes | 48 | 3 | 2 | 0.64 | 0.51 | 0.32 |
| C | testing_S1_C | 3 haplotype classes | 48 | 21 | 3 | 0.87 | 0.68 | 0.58 |
| D | testing_S1_D | 4 haplotype classes | 48 | 31 | 4 | 0.94 | 0.77 | 0.70 |
| E | testing_S1_E | lowest frequency evenness | 48 | 31 | 4 | 0.86 | 0.73 | 0.62 |
| F | testing_S1_F | lower frequency evenness | 48 | 31 | 4 | 0.89 | 0.75 | 0.66 |
| G | testing_S1_G | higher frequency evenness | 48 | 31 | 4 | 0.92 | 0.76 | 0.78 |
| H | testing_S1_H | equal frequencies | 48 | 31 | 4 | 0.94 | 0.77 | 0.70 |
