# Supplementary material for: Haplotype network branch diversity, a new metric combining genetic and topological diversity to compare the complexity of haplotype networks
Source: PLoS One. 2021 Jun 30;16(6):e0251878. doi: 10.1371/journal.pone.0251878 (PMC8244886; doi:10.1371/journal.pone.0251878)
Supplement: S2 Table — (DOCX) [file pone.0251878.s004.docx]

**S2 Table. Properties of the branch diversity (*Bd*) metric**. Each row represents a separate haplotype network. Columns show the number of haplotype classes (*nHc*), number of individuals per haplotype class (*niHc*), total number of individuals (*n*), and branch diversity (*Bd*). Property 4 is explained separately in S3 Table. NA, metric cannot be calculated for a single individual.

|  | ***nHc*** | ***niHc*** | ***n*** | ***Bd*** |
| --- | --- | --- | --- | --- |
|  |  |  |  |  |
| **Property 1.**  *Bd*=0 when there is only one haplotype class (*Hc*)regardless of its frequency (*niHc*). | 1 | 2 | 2 | 0 |
|  | 1 | 3 | 3 | 0 |
|  | 1 | 100 | 100 | 0 |
|  | 1 | 10000000 | 10000000 | 0 |
|  |  |  |  |  |
| **Property 2.**  *Bd* reaches the theoretical value of 1 when every individual forms a different haplotype class (i.e. with a unique number of branches). | 1 | 1 | 1 | *NA |
|  | 2 | 1 | 2 | 1 |
|  | 3 | 1 | 3 | 1 |
|  | 4 | 1 | 4 | 1 |
|  | 10 | 1 | 10 | 1 |
|  | 20 | 1 | 20 | 1 |
|  | 50 | 1 | 50 | 1 |
|  | 100 | 1 | 100 | 1 |
|  |  |  |  |  |
| **Property 3.**  *Bd* increases with increasing number of haplotype classes. | 1 | 8 | 8 | 0 |
|  | 2 | 8 | 16 | 0.533 |
|  | 3 | 8 | 24 | 0.696 |
|  | 4 | 8 | 32 | 0.774 |
|  | 10 | 8 | 80 | 0.911 |
|  | 20 | 8 | 160 | 0.956 |
|  | 50 | 8 | 400 | 0.982 |
|  | 100 | 8 | 800 | 0.991 |
|  | 1000 | 8 | 8000 | 0.999 |
|  | 10000 | 8 | 80000 | 0.999 |
|  | 100000 | 8 | 800000 | 0.999 |
|  | 1000000 | 8 | 8000000 | 0.999 |
|  | 10000000 | 8 | 80000000 | 0.999 |
|  | 100000000 | 8 | 800000000 | 0.999 |
|  | 1E+09 | 8 | 8E+09 | 0.999 |
|  | 1E+10 | 8 | 8E+10 | 0.999 |
|  |  |  |  |  |
|  | 1 | 1000 | 1000 | 0 |
|  | 2 | 1000 | 2000 | 0.500 |
|  | 3 | 1000 | 3000 | 0.667 |
|  | 4 | 1000 | 4000 | 0.750 |
|  | 10 | 1000 | 10000 | 0.900 |
|  | 20 | 1000 | 20000 | 0.950 |
|  | 50 | 1000 | 50000 | 0.980 |
|  | 100 | 1000 | 100000 | 0.990 |
|  | 1000 | 1000 | 1000000 | 0.999 |
|  | 10000 | 1000 | 10000000 | 0.999 |
|  | 100000 | 1000 | 100000000 | 0.999 |
|  | 1000000 | 1000 | 1E+09 | 0.999 |
|  | 10000000 | 1000 | 1E+10 | 0.999 |
|  | 100000000 | 1000 | 1E+11 | 0.999 |
|  | 1E+09 | 1000 | 1E+12 | 0.999 |
|  | 1E+10 | 1000 | 1E+13 | 0.999 |
|  |  |  |  |  |
| **Property 5.** *Bd* decreases with increasing number of individuals, n, unless these add new haplotype classes. | 5 | 1 | 5 | 1.000 |
|  | 5 | 2 | 10 | 0.889 |
|  | 5 | 3 | 15 | 0.857 |
|  | 5 | 4 | 20 | 0.842 |
|  | 5 | 5 | 25 | 0.833 |
|  | 5 | 10 | 50 | 0.816 |
|  | 5 | 100 | 500 | 0.802 |
|  | 5 | 10000 | 50000 | 0.800 |
|  | 5 | 1000000 | 5000000 | 0.800 |
|  | 5 | 1E+10 | 5E+10 | 0.800 |
|  |  |  |  |  |
|  | 10 | 1 | 10 | 1.000 |
|  | 10 | 2 | 20 | 0.947 |
|  | 10 | 3 | 30 | 0.931 |
|  | 10 | 4 | 40 | 0.923 |
|  | 10 | 5 | 50 | 0.918 |
|  | 10 | 10 | 100 | 0.909 |
|  | 10 | 100 | 1000 | 0.901 |
|  | 10 | 10000 | 100000 | 0.900 |
|  | 10 | 1000000 | 10000000 | 0.900 |
|  | 10 | 1E+10 | 1E+11 | 0.900 |
