# Supplementary material for: Haplotype network branch diversity, a new metric combining genetic and topological diversity to compare the complexity of haplotype networks
Source: PLoS One. 2021 Jun 30;16(6):e0251878. doi: 10.1371/journal.pone.0251878 (PMC8244886; doi:10.1371/journal.pone.0251878)
Supplement: S3 Table — (DOCX) [file pone.0251878.s005.docx]

**S3 Table.** **Branch diversity (*Bd*) property 4.** Hypothetical haplotype networks that show how *Bd* increases as the frequency (number of individuals) among haplotype classes (*niHc*) evens out. The breakdown illustrates seven networks, all with 240 individuals and 6 haplotype classes (of any given number of branches), and with the frequency among haplotype classes becoming more even from network 1 (highly skewed) to 7 (even frequencies). Columns indicate network, haplotype class (*Hc*), frequency or number of individuals per haplotype class (*niHc*), total number of individuals (*n*), and branch diversity (*Bd*).

|  | **Network** | ***Hc*** | ***niHc*** | ***n*** | ***Bd*** |
| --- | --- | --- | --- | --- | --- |
| **Property 4.** *Bd* increases with higher frequency-evenness among haplotype classes. |  |  |  |  |  |
|  | 1 | a | 194 | 240 | 0.336 |
|  |  | b | 20 | 240 |  |
|  |  | c | 15 | 240 |  |
|  |  | d | 5 | 240 |  |
|  |  | e | 3 | 240 |  |
|  |  | f | 3 | 240 |  |
|  |  |  |  |  |  |
|  | 2 | a | 188 | 240 | 0.375 |
|  |  | b | 20 | 240 |  |
|  |  | c | 15 | 240 |  |
|  |  | d | 5 | 240 |  |
|  |  | e | 6 | 240 |  |
|  |  | f | 6 | 240 |  |
|  |  |  |  |  |  |
|  | 3 | a | 128 | 240 | 0.601 |
|  |  | b | 80 | 240 |  |
|  |  | c | 15 | 240 |  |
|  |  | d | 5 | 240 |  |
|  |  | e | 6 | 240 |  |
|  |  | f | 6 | 240 |  |
|  |  |  |  |  |  |
|  | 4 | a | 100 | 240 | 0.703 |
|  |  | b | 80 | 240 |  |
|  |  | c | 15 | 240 |  |
|  |  | d | 15 | 240 |  |
|  |  | e | 15 | 240 |  |
|  |  | f | 15 | 240 |  |
|  |  |  |  |  |  |
|  | 5 | a | 60 | 240 | 0.816 |
|  |  | b | 60 | 240 |  |
|  |  | c | 30 | 240 |  |
|  |  | d | 30 | 240 |  |
|  |  | e | 30 | 240 |  |
|  |  | f | 30 | 240 |  |
|  |  |  |  |  |  |
|  | 6 | a | 60 | 240 | 0.828 |
|  |  | b | 36 | 240 |  |
|  |  | c | 36 | 240 |  |
|  |  | d | 36 | 240 |  |
|  |  | e | 36 | 240 |  |
|  |  | f | 36 | 240 |  |
|  |  |  |  |  |  |
|  | 7 | a | 40 | 240 | 0.837 |
|  |  | b | 40 | 240 |  |
|  |  | c | 40 | 240 |  |
|  |  | d | 40 | 240 |  |
|  |  | e | 40 | 240 |  |
|  |  | f | 40 | 240 |  |
